# The effect of rural-to-urban migration on renal function in an Indian population: cross-sectional data from the Hyderabad arm of the Indian Migration Study

**DOI:** 10.1186/1471-2369-14-240

**Published:** 2013-10-31

**Authors:** Phillippa K Bailey, Charles RV Tomson, Sanjay Kinra, Shah Ebrahim, KV Radhakrishna, Hannah Kuper, Dorothea Nitsch, Yoav Ben-Shlomo

**Affiliations:** 1 The Richard Bright Renal Unit, Southmead Hospital, Bristol BS10 5NB, UK; 2 London School of Hygiene and Tropical Medicine, London WC1E 7HT, UK; 3 National Institute of Nutrition, Hyderabad, India; 4 School of Social and Community Medicine, University of Bristol, Canynge Hall, Bristol BS8 2PS, UK

## Abstract

**Background:**

Urban migration is associated with an increased risk of hypertension, obesity and diabetes in Indian migrants. This study assessed the relationship between internal migration and renal function in the Hyderabad arm of the Indian Migration Study.

**Methods:**

We assessed 841 subjects; urban non-migrants (n = 158), urban migrants (n = 424) and rural non-migrants (n = 259). Muscle mass was ascertained from DXA scanning. We derived urban life years for urban migrants and rural non-migrants. Multivariable linear regression was used to examine the association between tertiles of urban life years and 4-variable MDRD eGFR using Stata 11.

**Results:**

Mean eGFR was lower in urban non-migrants and urban migrants compared to rural non-migrants. The prevalence of CKD 3-5 was higher in the rural non-migrant population (5.0%) than in the urban non-migrant populations (2.5%) due to a negatively skewed distribution of eGFR in rural non-migrants. As urban life years increased, eGFR declined (p = 0.008) though there was no obvious dose response effect. After adjustment for muscle mass, the association was attenuated and the trend was consistent with chance (p = 0.08). Further adjustment for vascular risk factors weakened the association to a small degree (p = 0.11).

**Conclusions:**

The high prevalence of reduced eGFR in rural areas requires further research. Urbanization was associated with reduced eGFR. This association appears mostly to be due to higher muscle mass with a small contribution from adverse vascular disease risk factors.

## Background

In comparison to host populations, migrants are more frequently subject to hypertension, obesity and chronic conditions such as diabetes [1]. Migrants’ ill-health and unfavourable risk profiles may worsen with increasing duration of stay [1]. In addition, urban populations have higher levels of cardiometabolic risk factors than rural populations [2-5] and rural to urban migration has been shown to be associated with an accruement of these risk factors [6,7]. Trends of increased obesity and diabetes among international South Asian migrants are well documented [8,9] and more recently have been demonstrated among internal rural-urban migrants in India [6,7].

Studies of renal function among South Asian populations living in the UK have shown higher acceptance rates for renal replacement treatment [10], which is probably secondary to a higher incidence of end-stage renal failure. This may be partly due to a higher prevalence of Type 2 diabetes and an increased risk of renal failure as a complication [11] but it is difficult in the UK to distinguish between the health effects of migration, and socio-cultural and ethnic differences in an epidemiological study. We have studied the effect of rural-to-urban migration on renal function within India. To date there has been no previous work published in this area.

Until recently, information on the prevalence and incidence of Chronic Kidney Disease (CKD) in India was limited to small studies, comparisons between which were limited by differences in study populations, methods of measuring or estimating renal function and definitions of CKD [12-14]. The Indian Renal Registry published its first report in 2012. The most common identifiable causes of CKD were diabetes, glomerulonephritis and hypertension [15]. ‘Unknown aetiology’ was the second most common ‘diagnosis’ after diabetes. It is not possible to determine from existing registry data whether there are differences in CKD prevalence between urban and rural areas.

The aim of this study was to look at differences in estimated Glomerular Filtration Rate (eGFR) between rural non-migrants, urban non-migrants and rural-to-urban migrants (urban migrants) within India, and to understand the reasons behind any differences observed. Differences in muscle mass between urban and rural populations may result in differences in creatinine-based eGFR. In this study we had the benefit of whole body Dual-energy X-ray Absorptiometry (DXA) scans and were therefore able to adjust findings for measured muscle mass.

It was hypothesized that urbanization would have a negative effect on eGFR, and that those living in an urban area, would have worse renal function, represented by a lower eGFR, than rural non-migrants. We expected the effects of urbanization to accrue and that in urban migrants, controlling for age, the greater the time spent in an urban environment, the lower eGFR. We expected the effect of urbanization to be the result of increased vascular disease and cardiometabolic risk factors.

## Methods

### Ethics statement

Ethics committee approval was obtained from the All India Institute of Medical Sciences Ethics Committee, reference number A-60/4/8/2004.

Using the framework of a cardiovascular risk factor screen study conducted in factories in north, central and south India [16] a sib-pair comparison study was designed. Details of the design have been reported elsewhere [17]. The original study was based in four Indian factories (Lucknow, Hindustan Aeronautics Ltd; Nagpur, Indorama Synthetics Ltd; Hyderabad, Bharat Heavy Electricals Ltd; and Bangalore, Hindustan Machine Tools Ltd). Factory workers and their co-resident spouses were recruited if they were urban migrants using employer records as the sampling frame. Each migrant worker and spouse invited one non-migrant full sibling of the same sex, closest to them in age still residing in their rural place of origin. A 25% random sample of non-migrants was also invited to participate. Information sheets were translated into local languages, signed (thumb print accepted), and consent obtained. Field work began in March 2005 and was completed by December 2007.

The Hyderabad arm of the Indian Migration Study is a more detailed follow-up study of the original subject from the Hyderabad centre. Participants were invited to a screening clinic at the National Institute of Nutrition between January 2009 and December 2010.

### Rural-urban status

Attempts were made to correct discrepancies in migrant status where the data allowed. Participants were excluded if mislabelling errors could not be corrected (eg participants had been labelled as urban non-migrants with no urban years recorded). In total 76 individuals were excluded due to discrepancies between urban life years and original migrant status.

### Clinic measures

Participants were interviewed and data collected on demographic factors including socioeconomic status by using a subset of 12 of 27 questions from the Standard of Living Index (SLI), a household level asset-based scale devised for Indian surveys [18]. These comprised house type, house ownership, toilet facility, source of lighting, source of drinking water, car or tractor, moped or scooter, telephone, refrigerator, television, bicycle, radio, clock or watch, and weighted to give a maximum score of 33. Weights of items for the SLI were developed by the International Institute of Population Sciences in India. On the basis of this score, individuals were classified as having a ‘low’ (0-7), ‘middle’ (8-12) or ‘high’ (13-33) standard of living. Smoking was assessed as positive if individuals currently/actively smoked or chewed tobacco. Few individuals reported previous but not current smoking.

A diagnosis of Coronary Heart Disease (CHD) and Stroke was made by self-report of a doctor diagnosis.

Weight was measured twice to the nearest 0.1 kg without shoes using digital Seca scales (http://www.seca.com). Standing height was measured twice without shoes using a portable stadiometer (Leicester height measure; Chasmors Ltd, Camden, London, UK). The participant stood erect with his or her head in the Frankfort plane, and a gentle upward pressure was applied under the mastoid. Waist circumference was measured twice to the nearest mm using a material tape measure at the narrowest point of the waist between the ribs and the iliac crest. The average of the two values was used in the analysis. Body Mass Index (BMI) was calculated as weight(kg)/height(m)^2^. We used a validated oscillometric device (OMRON M5-I; Omron, Matsusaka Co, Japan) to measure blood pressure in the sitting position with appropriate cuff sizes. We took three measures, 2-3 minutes apart, and averaged the measures for analyses. A diagnosis of Hypertension was made if the average of three systolic blood pressure (BP) measurements was ≥140 mmHg, if the average of three diastolic BP measurements was ≥90 mmHg or if there was report of a doctor diagnosis of Hypertension [19].

### Laboratory assays and anthropometric measurements

Participants were asked to attend fasting and the time of their last meal was recorded. Creatinine analysis was performed using the rate-blanked and compensated Jaffe method for creatinine estimation on a COBAS C311 autoanalyzer from ROCHE. The calibraton for this assay is traceable to isotope dilution mass spectrometry (IDMS), that is, the method used is calibrated to the single standardized serum creatinine using reference materials traceable to the primary reference material at the National Institute of Standards, based on IDMS. The Cardiac Biochemistry Lab, AIIMS, is part of the UK National External Quality Assessment Scheme (http://www.ukneqas.org.uk) to quality assure its assays.

A diagnosis of diabetes was made using the World Health Organization (WHO) fasting plasma glucose criterion of >7.0 mmol/l [20] or report of a doctor diagnosis of diabetes. Homeostasis model assessment (HOMA) scores to estimate insulin resistance were calculated from fasting blood glucose and serum insulin levels using a standard formula of plasma glucose (mmol/l) × plasma insulin (mU/l)/22.5, on the basis of the original approach [21]. HOMA has been validated by comparison with biochemical markers of insulin resistance in healthy Indian people, yielding moderate correlations [22]. Low values indicate high insulin sensitivity, whereas high values indicate low insulin sensitivity (insulin resistance).

Whole body DXA scans were performed on a Hologic DXA machine (Discovery A model) (91% of scans) or a Hologic QDR 4500 Elite machine (http://www.hologic.com) (9% of scans). Participants were asked to remove jewellery and to change in to light clothing. During the scan, the participant was asked to lie supine on the scanning bed with their arms at their sides. Whole body scans were visually checked for artefacts and those with major artefacts were removed from the analyses. For quality assurance, a spine phantom was scanned every day to check for acceptable ranges. DXA exploits the attenuation of two photo energies to determine the mass of mineral, fat and lean in the body. Wang et al found that lean mass in the extremities represents approximately 75% of the total body skeletal muscle mass [23]. Thus total skeletal muscle mass was determined from the lean body mass of all four extremities multiplied by 1.33. Body Surface Area (BSA) was calculated from height and weight with the Mosteller equation [24]. Following previous work to examine the performance of various creatinine-based renal function estimating formulae [25], we estimated renal function using the IDMS standardized Modification of Diet in Renal Disease (MDRD) formula:

$$\begin{array}{l} \mathrm{eGFR}=175\times {\left(\mathrm{Serum}\;\mathrm{creatinine}\left(\mathrm{mg}/\mathrm{dl}\right)\right)}^{-1.154}\times \mathrm{Ag}e^{-0.203}\\ \;\times \left[0.742\;\mathrm{if}\;\mathrm{female}\right]=\mathrm{ml}/\min/1.73m^{2} \end{array}$$

CKD was classified according to the KDOQI guidelines [26]. As information on albuminuria or proteinuria was unavailable, stages 1 and 2 were not identified. We defined an eGFR <60 ml/min/1.73 m^2^ as ‘CKD stages 3-5’. In clinical practice, the diagnosis of CKD requires evidence of reduced function for ≥3 months, however it is accepted in epidemiological studies that a single estimate of GFR is used for a diagnosis of CKD [27].

### Statistical analyses

Linear regression and Pearson’s correlation coefficient were used to investigate the relationship between eGFR and age within each group (rural non-migrants, urban migrants and urban non-migrants). We undertook sex-stratified analyses as well as combined analyses after formally testing for any sex interaction.

After excluding urban non-migrants, we generated tertiles from urban life years (Tertile 1: 0 years (n = 228) Tertile 2: 0.9 to 28.8 years (n = 228), Tertile 3: 28.8 to 51.8 years (n = 227)) and proportion of life in an urban area (Tertile 1: 0, Tertile 2: 0.01 to 0.57, Tertile 3: 0.58 to 0.98). It was possible to generate true tertiles as a small number of ‘rural non-migrants’ although primarily rural dwellers had spent some time living in urban areas (<0.4 proportion of life and fewer than 22 years). We performed multivariable linear regression (with tertiles as dummy variables) to calculate the regression coefficient between eGFR and urban life years with rural non-migrants as the baseline comparator group. The analysis was performed both unadjusted and adjusted for vascular disease risk factors and body composition: age, HOMA-IR, diabetes, smoking, cholesterol, BMI, hypertension and DXA measured lean muscle mass.

As the study design includes related individuals, who are not truly independent, we performed our regression models using robust standard errors to allow for any family clustering effect. This provides larger p-values and more conservative standard errors. A sensitivity analysis was repeated with ‘proportion of life spent resident in an urban area’. All statistical analyses were performed using Stata version 11.

## Results

**Figure 1:**
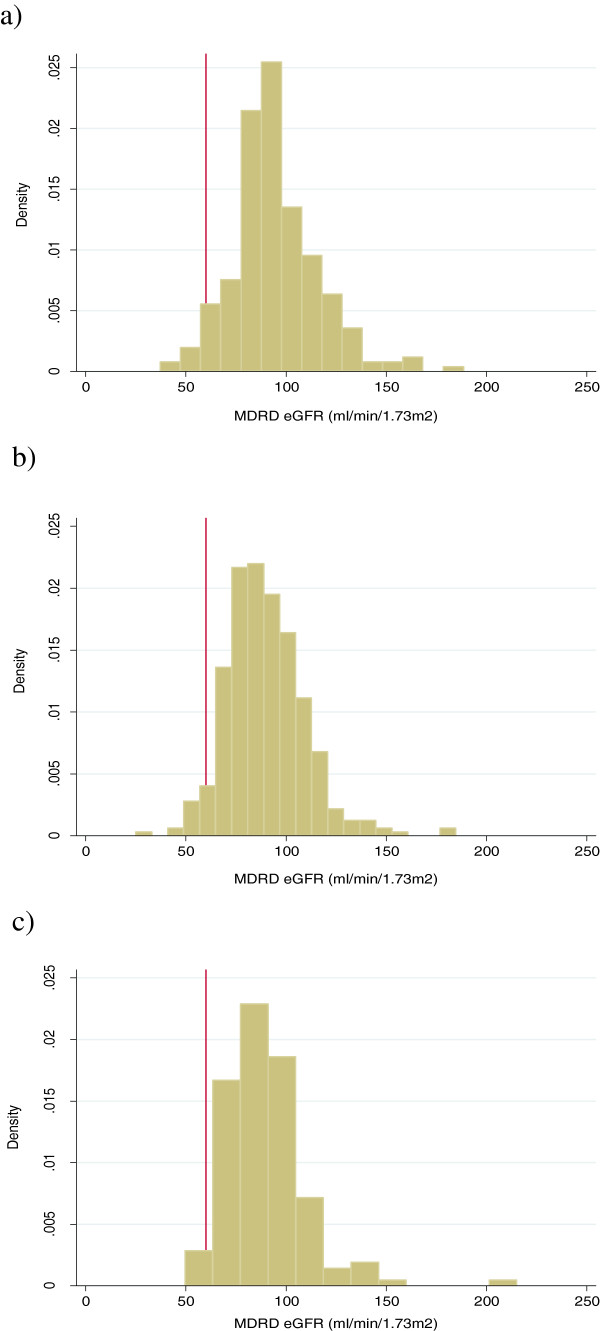
**Histograms of MDRD eGFR for rural non-migrants, rural-urban migrants and urban non-migrants. a)** Rural non-migrants. **b)** Rural-urban migrants. **c)** Urban non-migrants.

917 individuals were eligible for analysis. 76 individuals were excluded from analyses due to discrepancies between urban life years and original migrant status. The study sample comprised 841 adult subjects (mean age 48.4 ± S.D. 8.3 years) and roughly equal proportions of men and women in the overall sample and in the subgroups (chi2 p value 0.26) (Table 1). The BMI of rural non-migrants was 3.4 kg/m^2^ lower than that of urban non-migrants (95% CI 3.2-3.6). Mean muscle mass in rural non-migrants was 1.2 kg (95% CI 1.0-1.3) less than in urban non-migrants. Smoking was more prevalent amongst rural non-migrants (17.8%) compared with urban migrants (8.5%) and urban non-migrants (7.6%). The prevalence of diabetes and hypertension was lowest in the rural non-migrants. Mean eGFR as estimated by MDRD was lower in urban non-migrants and urban migrants compared to rural non-migrants. Rural non-migrants had the highest mean eGFR. However, the prevalence of CKD stages 3-5 was higher in the rural non-migrant population (5.0%) than in the urban migrant population (4.2%) and the urban non-migrant population (2.5%). The higher prevalence of those with eGFR < 60 ml/min/1.73 m^2^ in the rural group is explained by the histogram showing a negatively skewed distribution (Figure 1). The number of people with CKD stages 3-5 was very small in all groups and the proportions were similar for all groups (p value = 0.20).

**Table 1:** Baseline characteristics of Hyderabad arm of the Indian migration study stratified by migration status

	**All**	**Rural non-migrants**	**Rural to urban migrants**	**Urban non-migrants**
No of observations	841	259	424	158
% female	47.1	43.6	47.4	51.9
**Mean (± S.D.)**				
Age (years)	48.4 (±8.3)	47.5 (±10.3)	49.4 (±6.7)	46.9 (±8.5)
Weight (kg)	65.4 (±11.9)	61.0 (±12.6)	66.8 (±10.7)	68.9 (±11.9)
Height (cm)	159.0 (±8.7)	159.0 (±8.7)	159.2 (±8.5)	158.7 (±9.2)
BMI (kg/m^2^)	25.9 (±4.4)	24.1 (±4.5)	26.4 (±3.9)	27.5 (±4.7)
BSA (m^2^)	1.69 (±0.18)	1.63 (±0.19)	1.71 (±0.16)	1.74 (±0.17)
Muscle Mass (kg)	24.1 (±5.4)	23.4 (±5.4)	24.4 (±5.4)	24.6 (±5.2)
Muscle mass (kg per 1.73 m^2^)	24.4 (±3.6)	24.5 (±3.7)	24.4 (±3.7)	24.3 (±3.5)
SBP (mmHg)	122 (±16)	121 (±17)	122 (±16)	121 (±16)
DBP (mmHg)	81 (±10)	79 (±10)	82 (±10)	81 (±9)
Creatinine (μmol/l)	73.1 (±17.7)	72.1 (±17.8)	73.7 (±18.4)	73.3 (±15.7)
Total cholesterol	4.9 (±1.0)	4.8 (±1.1)	4.9 (±1.0)	4.8 (±1.0)
HOMA-IR score	2.74 (± 5.30)	1.68 (± 1.94)	3.22 (± 6.21)	3.21 (±6.12)
MDRD eGFR (ml/min/1.73 m^2^)	91.3 (±20.5)	94.7 (±21.7)	89.8 (±19.6)	89.7 (±20.1)
	[95% CI 89.9-92.7]	[95% CI 91.9-97.4]	[95% CI 87.8–91.7]	[95% CI 86.5–93.0]
**Number (%)**				
SES–Low	15 (1.8%)	14 (5.4%)	1 (0.2%)	0 (%)
SES–Middle	43 (5.1%)	39 (15.1%)	2 (0.5%)	2 (1.3%)
SES–High	783 (93.1%)	206 (79.5%)	421 (99.3%)	156 (98.7%)
PMH diabetes	148 (17.6%)	26 (10%)	89 (21.0%)	33 (20.9%)
Diabetes	207 (24.6%)	44 (17.0%)	119 (28.1%)	44 (27.8%)
Hypertension	300 (35.7%)	73 (28.2%)	161 (38.0%)	66 (41.8%)
Smoking status–current	94 (11.1%)	46 (17.8%)	36 (8.5%)	12 (7.6%)
Vascular disease (CHD and Stroke)	37 (4.4%)	7 (2.7%)	24 (5.7%)	6 (3.8%)
MDRD–CKD 3-5	35 (4.1%)	13 (5.0%)	18 (4.2%)	4 (2.5%)

BMI–Body Mass Index; BSA–Body Surface Area; SBP–systolic blood pressure; DBP–diastolic blood pressure; HOMA-IR–Homeostatic Model Assessment–Insulin Resistance; MDRD eGFR–Modification of Diet in Renal Disease estimated Glomerular Filtration Rate; SES–Socio–Economic Status; PMH–Past Medical History; CHD–Coronary Heart Disease; CKD–Chronic Kidney Disease.

The relationship between eGFR and age was examined with linear regression in each group (rural non-migrants, urban migrants and urban non-migrants). eGFR declined with increasing age in all groups and no heterogeneity was observed between groups (Additional file 1: Table S1 + Additional file 1: Figure S1).

Linear regression analysis revealed that at any given age, the mean eGFR was 5.9 ml/min/1.73 m^2^ (p = 0.007, 95% CI −10.1 to −1.6) lower in urban non-migrants compared to rural non-migrants, and 4.0 ml/min/1.73 m^2^ (p = 0.011, 95% CI −7.0 to −0.9) lower in urban migrants compared to rural non-migrants.

The analysis was then restricted to rural non-migrants and urban migrants to investigate the association of urban life years with eGFR in migrants. The relationship was examined within tertiles generated from urban life years compared to rural non-migrants as the baseline comparator group, and sequentially adjusted results are shown including adjustments for known vascular disease risk factors and body composition, including measured muscle mass (Table 2). Results were similar for men and women. As the number of urban life years increased, eGFR declined though there was no obvious dose response effect (eGFR for tertiles 2 and 3 compared to baseline;−4.72,−4.67 ml/min/1.73 m^2^; p value for trend = 0.008) suggesting that the relationship may plateau rather than show a linear dose-response pattern. After adjustment for muscle mass, the associations were attenuated, suggesting limited power (p value for trend = 0.08). Further adjustment for HOMA-IR, diabetes, smoking, cholesterol, hypertension and BMI weakened the association further to a small degree, although the estimates remained stable in size, the confidence intervals were wide (p value for trend = 0.11).

**Table 2:** **Relationship between urban life years and eGFR (ml/min/1.73 m**^**2**^**)**

	**Adjusted for age** **Difference in eGFR between migrants and non-migrants (reference group tertile 1) in ml/min/1.73 m**^**2**^			**Adjusted for age and muscle mass** **Difference in eGFR between migrants and non-migrants (reference group tertile 1) in ml/min/1.73 m**^**2**^			Adjusted for age, muscle mass and cardiometabolic risk factors* Difference in eGFR between migrants and non-migrants (reference group tertile 1) in ml/min/1.73 m^**2**^		
Urban life years	Men and women	Men	Women	Men and women	Men	Women	Men and women	Men	Women
Tertile 2	−4.72	−5.67	−4.65	−3.90	−4.02	−3.91	−3.35	−3.32	−4.11
	(-8.42,-1.03)	(-10.28,-1.05)	(-10.69,1.39)	(-7.62,-0.18)	(-8.92,0.87)	(-10.08,2.27)	(-7.11,0.41)	(-8.40,1.76)	(-10.14,1.91)
	p = 0.01	p = 0.02	p = 0.13	p = 0.04	p = 0.11	p = 0.21	p = 0.08	p = 0.20	p = 0.18
Tertile 3	−4.67	−4.87	−6.06	−3.07	−2.91	−3.78	−3.00	−2.79	−4.92
	(-8.42,-1.03)	(-10.28,-1.05)	(-10.69,1.39)	(-7.62,-0.18)	(-8.92,0.87)	(-10.08,2.27)	(-7.11,0.41)	(-8.40,1.76)	(-10.14,1.91)
	p = 0.01	p = 0.05	p = 0.03	p = 0.10	p = 0.25	p = 0.20	p = 0.12	p = 0.28	p = 0.10
p value for trend across tertiles of urban life-years	0.008	0.04	0.03	0.08	0.21	0.20	0.11	0.26	0.09

*Adjusted for HOMA-IR, diabetes, smoking, cholesterol, hypertension and BMI.

The analysis was repeated with ‘proportion of life spent resident in an urban area’ , and the same pattern found (data not shown).

## Discussion

This study examines the effect of urban migration on renal function in an Indian population. Urbanization appears to be associated with reduced MDRD–derived eGFR. This association appears in part explained by a higher muscle mass with a small contribution from adverse vascular risk factors. These findings suggest that any eGFR comparisons between rural areas and urban areas in India should adjust for muscle mass. The finding of higher muscle mass in urban residents may not have been expected. The physical activity of the rural non-migrants is likely to be greater than that of urban residents but this may be mitigated by nutritional factors such as dietary protein. It is possible that because our sample is relatively young and fit, physical activity levels may be similar at these ages.

In addition, there was a suggestion in this study that poor renal function may be more prevalent in rural areas than previously thought as evidenced by the surprisingly high prevalence of low eGFR.

### Rural risk factors for CKD

The prevalence of CKD stages 3-5 was actually higher in rural non-migrants than both urban migrants and urban non-migrants, although in all groups the number of individuals with CKD was very small. In our study group, smoking prevalence was higher in the rural than the urban population, but this alone is unlikely to fully explain the observed differences. Other factors may be contributing to an increased risk of CKD in rural areas in India. We are not the first to observe such a phenomenon; a similar study examining the prevalence of CKD in Thailand found that developed urban areas had lower prevalence of CKD compared to less-developed rural areas [28]. This was despite the prevalence of diabetes and hypertension being greater in the urban areas, as in our study. The authors speculated that endemic infection, sanitation and access to health care may have influenced this observed difference. Similar reports of high rates of renal disease in rural areas have been made in other South Asian populations. In Sri Lanka clusters of CKD have been reported in male farmers, possibly associated with dietary cadmium [29,30]. In India, a number of other factors influencing CKD in rural areas have been postulated, including environmental toxins such as herbal therapies, pesticides and contaminated drinking water, rice and fish [31,32]. In addition, the recently reported ‘epidemic’ of CKD in agricultural communities in Nicaragua raises a number of other possible infective and nephrotoxic causes, which may be relevant to the Indian setting [33]. Such observations challenge the assumption that CKD in South Asia is primarily because of urbanization and increased diabetes and hypertension.

### Urbanization and CKD

Clear evidence exists of urban life years being associated with a range of vascular risk factors [6,7]. This paper showed that there may also be an association with a decreasing eGFR with years spent in an urban environment, although some of this association may be explained in part by differences in muscle mass. This population is relatively young and fit, and the potential adverse effects of urban living may be more pronounced in a population of people with higher susceptibility to renal damage; those with advanced age and those with a greater number of comorbidities. In addition the early effects of obesity and diabetes mellitus are to cause hyperfiltration [34] and thus an ‘increase’ in eGFR if estimated using creatinine based formulae [35]. A study from the 1946 British Birth Cohort suggests that there may be a 20 year lag between the onset of being overweight and the detection of an increased prevalence of CKD [36]. One may therefore expect a substantive lag between first renal insult and actually observing reduced eGFR.

This study has a few limitations: *1) Cross-sectional data* As this study is cross-sectional, we can only infer that the observed differences represent declining eGFR with urbanization, and duration of urban life. *2) Creatinine based formulae for estimating renal function* In this population renal function was estimated, not measured. The MDRD formula performs less well at the extremes of renal function [27], a significant limitation of estimating rather than measuring renal function. However, a unique strength of this study is the ability of DXA scans to allow adjustment for differences in muscle mass. *3) Lack of chronicity data* In clinical practice, the diagnosis of CKD requires evidence of reduced function for ≥3 months. In this study we have relied on a single eGFR, an approach accepted in epidemiological studies [27], but one that tends to inflate the reported prevalence of CKD by ignoring creatinine fluctuation [37]. In addition, for CVD we have used self-reporting of a diagnosis which we recognise may underestimate the prevalence these conditions. *4) CKD stages 1-2* We do not have information regarding renal tract anatomy or urinalysis, and are therefore unable to make any comment regarding CKD stages 1-2. In order to study the timing of the development and progression of CKD this early stage data is essential. *5) Selection bias* Since the study only includes 259 rural non-migrants for 424 urban migrants, the included rural non-migrants, selected by their urban migrant siblings, may well be systematically different from those who didn't participate. 6) Non-generalisability The subjects in this study were sampled by virtue that the index case was a factory worker. Therefore one would need to be very cautious in extrapolating our observational data to the general population. However the purpose of this study was to test a specific hypothesis as regards to the health effects of migration rather than to generalise our observations to the total population (see recent paper on the issue of representativeness) [38].

## Conclusions

As stated, this study is to our knowledge, the first to examine the effect of urbanization on renal function in India. It supports the hypothesis that urbanization has an adverse effect on renal function, and that vascular risk factors such as diabetes and obesity are in part responsible for this difference, but differences in muscle mass also partly account for our observations. Further work is required to confirm this association is seen with measured renal function. Spline regression modelling could be used to try to better characterise at what stage after urban migration there is a slowing in the decline of renal function and how this relates to changes in other risk factors such as blood pressure and obesity. Follow-up data will be of interest. Given the unexpectedly high prevalence of CKD in our rural population, future research should also focus on risk factors for renal disease specific to rural populations.

## Competing interests

The authors declare that they have no competing interests.

## Authors’ contributions

SK SE YBS KR and HK contributed to study design. HK SK and SE managed the fieldwork, staff training and supervision of the data collection for the study. PB CT and YBS contributed to data analysis. PB CT YBS and DN contributed to data interpretation. PB wrote the first draft of the paper. CT YBS and DN contributed to the writing of the paper. All authors provided feedback on the manuscript and approved the submitted version.

## Pre-publication history

The pre-publication history for this paper can be accessed here:

http://www.biomedcentral.com/1471-2369/14/240/prepub

## Supplementary Material

Additional file 1: Table S1. Correlation and regression estimates between MDRD eGFR and age within different migrant groups, using robust standard errors in the models to allow for any sibling clustering effect. **Figure S1.** Variation of MDRD eGFR with age within rural non-migrants, rural-urban migrants and urban non-migrants. **a)** Rural non-migrants. **b)** Rural-urban migrants. **c)** Urban non-migrants. Click here for file
