# Supplementary material for: The effect of rural-to-urban migration on renal function in an Indian population: cross-sectional data from the Hyderabad arm of the Indian Migration Study
Source: BMC Nephrol. 2013 Oct 31;14:240. doi: 10.1186/1471-2369-14-240 (PMC4228419; doi:10.1186/1471-2369-14-240)
Supplement: Additional file 1: Table S1. — Correlation and regression estimates between MDRD eGFR and age within different migrant groups, using robust standard errors in the models to allow for any sibling clustering effect. Figure S1. Variation of MDRD eGFR with age within rural non-migrants, rural-urban migrants and urban non-migrants. a) Rural non-migrants. b) Rural-urban migrants. c) Urban non-migrants. [file 1471-2369-14-240-S1.doc]

**Table S1:** Correlation and regression estimates between MDRD eGFR and age within different migrant groups, using robust standard errors in the models to allow for any sibling clustering effect.

|  | R^2^ | Regression coefficient MDRD eGFR with age | 95% Confidence Intervals |
| --- | --- | --- | --- |
| Rural non-migrants | 0.07 | -0.55 | -0.81, -0.29 |
| Urban migrants | 0.08 | -0.83 | -1.09, -0.58 |
| Urban non-migrants | 0.12 | -0.82 | -1.23, -0.41 |

**Figure S1**: Variation of MDRD eGFR with age within rural non-migrants, rural-urban migrants and urban non-migrants

a) Rural non-migrants

b) Rural-urban migrants

c) Urban non-migrants
